# Asymptomatic Meconium Peritonitis Presenting as Inguinal Hernia in a Female Neonate

**Published:** 2013-10-01

**Authors:** Sivasankar Jayakumar, Laila Hatsell, Nitin Patwardhan

**Affiliations:** University Hospitals Leicester, Infirmary road, Leicester, LE15WW, UK

**Keywords:** Bilateral inguinal hernia, Incarcerated inguinal hernia, Antenatal perforation

## Abstract

Inguinal hernias in girls are often irreducible when they contain ovaries. Rarely the hernial sacs may have unusual contents like vermiform appendix, uterus and urinary bladder. We report a case of a female infant who presented with bilateral irreducible inguinal hernias presumed to be due to ovaries. However at exploration, the hernial sacs contained bilaterally an omental mass with calcifications. Presence of mucin with meconium- laden macrophages in the mass on histology suggested an antenatal intestinal perforation. To the best of our knowledge no such case has been reported in a female neonate. We present this rare case and discuss the unusual findings and the outcome.

## INTRODUCTION

Most inguinal hernias in female infants are reducible and indirect; however, the hernia can become incarcerated. In the absence of bowel obstruction, ovaries are the most likely content seen in female infants [1]. We report a case of a female infant who presented with bilateral irreducible inguinal hernias presumed to be due to ovaries. However at prompt exploration, the hernial sacs contained bilaterally an omental mass with calcifications. Histopathological examination confirmed the omental mass to be consistent with meconium peritonitis. We report this unusual case and herein discuss the unusual findings and the outcome. 

## CASE REPORT

A preterm female baby born at 30 week gestational age was delivered by emergency caesarean section in view of maternal pre-eclampsia. Antenatal foetal scans did not reveal any abnormality. She was initially intubated for respiratory distress syndrome but was self ventilating by day 9 of life. She passed meconium on day 2 of life and was opening her bowels regularly. Abdominal and genitalia examination were unremarkable. Feeds were commenced on day 9 and were established slowly. She had an AXR to check the position of the umbilical catheter on day 2 of life and this did not reveal any abnormality.

At 6 weeks of age she was referred to us with bilateral incarcerated inguinal hernias. She had no evidence of bowel obstruction and clinically there were firm masses on both groins suggesting ovary as the most likely content. Bilateral inguinal herniotomy was carried out promptly. However, Intra-operatively on either side the inguinal hernial sacs contained omentum attached to an irregular, grey coloured mass with nodules distally. This mass was excised, healthy omentum returned and a standard herniotomy carried out on both sides. Post-operatively she recovered well and was discharged the following day. Histopathological examination confirmed the excised mass to be made up of omentum with areas of dystrophic calcification reminiscent of the saponification with mucin and meconium-laden macrophages; consistent with antenatal perforation of intestine (Fig. 1). At 3 months of follow-up, she remains well and opening her bowels regularly. 

**Figure:**
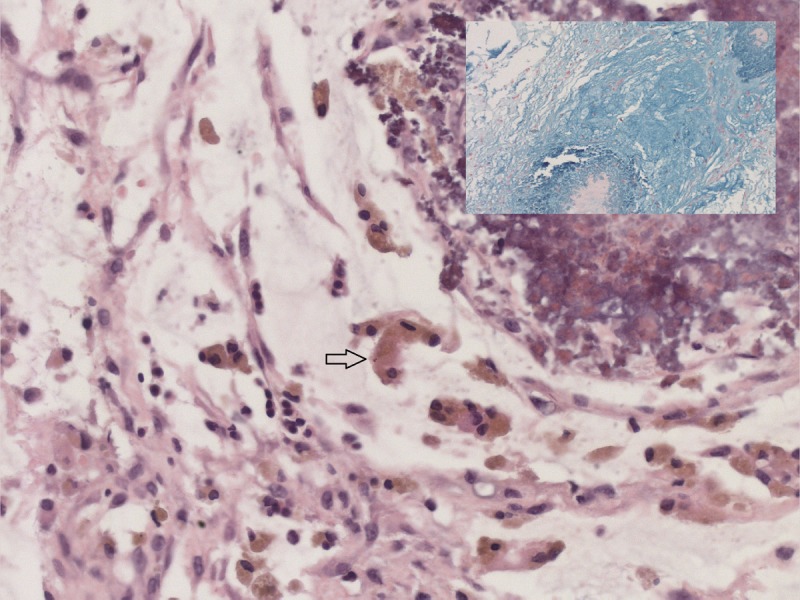
Figure 1: Histological images of omental mass excised during inguinal herniotomy in our patient displaying omentum with meconium-laden macrophages (arrow), Haemotoxylin and Eosin stain, magnification X40; [Inset displaying mucin, stained with Alcian blue, magnification X10]

## DISCUSSION

Incarcerated inguinal hernias in female infants are commonly due to herniated ovaries in the absence of bowel obstruction. Ovaries are reported in almost 90 – 100% of girls less than 1 year of age with incarcerated inguinal hernias [1]. Although, the ovary is not at great risk of strangulation, there is an increased risk of torsion [27%] and therefore prompt surgery is recommended [1]. Given such a high incidence of irreducible ovaries in female infants with incarcerated inguinal hernias, it is extremely rare to find hernial contents other than ovaries.

Meconium laden omental mass in inguinal hernias have been reported in many male infants [2-4], however not so in female infants. In our patient, meconium laden grey omental masses were noted to herniate through the inguinal canal bilaterally. The mass was excised off a normal looking omentum proximally on both sides and herniotomy carried out. The only case report in literature on a female infant similar to such a presentation was a term infant born with a right sided labial swelling consistent with meconium hydrocele within the canal of Nuck [5]. In the absence of signs of an acute bowel perforation, asymptomatic antenatal bowel perforation with meconium peritonitis should be considered in such cases.

Meconium peritonitis is a sterile process resulting from in utero intestinal perforation. Any pathology causing bowel obstruction in utero can lead to meconium peritonitis; however the incidence is low and is reported almost as 1 in 30000 [6]. Antenatal bowel obstruction is commonly seen with intestinal atresias, but can be transitory as seen in cystic fibrosis with meconium ileus. In utero ischemia of bowel has also been reported to cause bowel perforation and subsequent meconium peritonitis [7]. 

Antenatal ultrasound can reveal findings suggestive of meconium peritonitis. Kamata et al described three types of meconium peritonitis based on ultrasound findings: Type 1: Meconium ascites, Type 2: Giant pseudocyst, Type 3: Peritoneal calcification and small pseudocyst. In their same series of 20 patients, intra-abdominal calcifications were seen in only 25% of the patients [8] suggesting that not all cases of meconium peritonitis have findings on antenatal ultrasound scans and therefore antenatal diagnosis of meconium peritonitis can be missed. In our patient routine antenatal ultrasound scans failed to reveal any evidence of meconium peritonitis. 

Clinical manifestation of meconium peritonitis after birth depends on the extent of peritonitis and the underlying abnormality of bowel. It can present as giant meconium pseudocysts [9] or meconium stained inflammatory masses. A patient with an unusual sequela of meconium peritonitis involving massive extension of a processes vaginalis to the contralateral anterior abdominal wall has been reported [4]. However, meconium peritonitis does not always produce significant inflammation and may resolve spontaneously without any sequelae. Estroff et al reported 2 cases of meconium peritonitis detected antenatally that resolved spontaneously without any sequelae [10]. 

Ekinzi et al reported a male infant who was incidentally found to have meconium mass at bilateral inguinal hernia surgery [2]. The abdomen was explored and loose small bowel adhesions with viable small bowel were noted. They recommended that with such findings in the absence of bowel obstruction, further abdominal exploration is unnecessary. In our patient, we did not explore the abdomen and performed a routine herniotomy excising the herniated omental mass. Our patient has remained well since the surgery and to date has not had any complications. 

In conclusion, meconium peritonitis can be asymptomatic and can present as omental mass herniating through the inguinal canal. Abdominal x-rays might not reveal intra-abdominal calcifications in all patients, as in our case. Clinically asymptomatic neonates with omental mass found incidentally at inguinal herniotomy can be managed with inguinal herniotomy alone and further exploration of bowel we believe is unnecessary. 

## Footnotes

**Source of Support:** Nil

**Conflict of Interest:** None

